# Protective role of glutathione in oxidative stress caused by cadmium and copper

**DOI:** 10.1192/j.eurpsy.2022.2298

**Published:** 2022-09-01

**Authors:** J. Jovanovic Mirkovic, G. Kocic, Z. Jurinjak, C. Alexopoulos

**Affiliations:** 1 The Academy of Applied Preschool Teaching and Health Studies, Biochemistry, Cuprija, Serbia; 2 Faculty of Medicine, Biochemistry, Nis, Serbia; 3 The Academy of Applied Preschool Teaching and Health Studies, Cuprija, Serbia, Medicine, Cuprija, Serbia

**Keywords:** Glutathione, cadmium, copper, oxidative stress

## Abstract

**Introduction:**

Cadmium is defined as one of the leading toxic industrial pollutants (Valko i sar., 2005). Although some products containing cadmium can be recycled, much of the pollution with this metal is the result of inadequate disposal and uncontrolled incineration of cadmium-containing waste (Jarup, 2003). Copper particles are released into the atmosphere from copper smelters and ore processing facilities, as well as from anthropogenic sources (use of pesticides, herbicides and fungicides). Oxidative stress occurs due to increased production of reactive oxygen species (Parkinson’s and Alzheimer’s disease) or reduced ability of cells to neutralize it through their internal antioxidants (eg mutation of the superoxide dismutase gene in amyotrophic lateral sclerosis).

**Objectives:**

The aim of this research was to examine the protective role of supplement, GSH, S-donor ligand, and in conditions of acute and chronic intoxication with sublethal doses of cadmium-II-chloride and copper II sulfate.

**Methods:**

After medial laparotomy albino rates Wistar soy, a 10% homogenate of brain tissue was made in an appropriate medium and an analysis of acid and alkaline DNase activity was performed (Kocić i sar., 2004; Kocić i sar., 1998).

**Results:**

This experiment demonstrated the beneficial role of GSH supplement that exhibit antioxidant character in preventing and reducing the adverse effects of acute and chronic cadmium and copper intoxication.

**Conclusions:**

Antioxidants prevent the formation of oxidative stress in the cell by reducing and stopping DNA damage and degradation, and thus represent potential scavengers of free radicals.

**Disclosure:**

No significant relationships.

